# The role of precautions: Organising a medical conference during COVID‐19 pandemic—Lessons from IADVL MIDDERMACON 2021

**DOI:** 10.1002/jvc2.32

**Published:** 2022-06-14

**Authors:** Ramesh Bhat, Suvidha D. Kamath, Nicole Sequeira, Sunil Raina, Ganesh S. Pai, Dandakeri Sukumar, Jacintha Martis

**Affiliations:** ^1^ Department of Dermatology Father Muller Medical College Mangalore Karnataka India; ^2^ Father Muller Medical College Mangalore Karnataka India; ^3^ Department of Community Medicine Dr. R P Government Medical College Tanda Himachal Pradesh India; ^4^ DERMA‐CARE Skin and Cosmetology Centre Mangalore Karnataka India

**Keywords:** conference protocol, offline conference, pandemic, safe precautionary measures, vaccine

## Abstract

**Background:**

The use of virtual platforms for clinical meetings has become the default approach during this pandemic era. Organising an offline conference during a pandemic is a challenge and is possible if the participating crowd is vaccinated and is willing to follow appropriate pandemic protocols.

**Objective:**

To determine the feasibility of conducting a conference among mostly vaccinated delegates using standard precautionary protocols.

**Methods:**

This study was conducted at IADVL MIDDERMACON 2021, held in Mangalore, India, in late October 2021, during the phase of decline of the Delta variant of SARS‐CoV‐2. The study population included all conference attendees, including support staff. Details were collected about their vaccination status, comorbidities, and mode of travel to the conference venue. An reverse‐transcription polymerase chain reaction (RT‐PCR) test was done randomly among the attendees for COVID‐19 infection. A post‐conference assessment and RT‐PCR tests were done at the end of 2 weeks to assess the occurrence of infections among study participants.

**Results:**

A total of 1744 people were present at the venue, of which 576 (33.03%) participated in the study. The percentage of fully vaccinated was 88.88% (512/576). The majority had taken the vaccine Covishield (manufactured by AstraZeneca), that is, 85.06% (490/576). Infection post the conference was reported in 0.195% (1/576).

**Conclusions:**

Holding large gatherings like medical conferences pose a challenge during a pandemic. However, to increase the benefits of the conference, it is advisable to hold them offline with vaccinated delegates, follow the advice of the conference organising committee, and practise safe precautionary measures.

## INTRODUCTION

The COVID‐19 pandemic led to the cancellation of on‐site meetings^1^ and caused a shift towards virtual conferences. While these fulfil specific needs and have advantages, most physicians consider virtual meetings less immersive and more overwhelming; furthermore, they offer less efficient networking opportunities^2^ and decreased achievement of educational objectives.^2^, ^3^

SARS‐CoV‐2 vaccines have been proven effective in protecting against infection and transmission and even more against severe disease and mortality.^4^, ^5^ With widespread vaccination, on‐site conferences are feasible, provided necessary precautions are taken, including mask use (N95 masks in particular), social distancing, and frequent hand sanitisation.^6^, ^7^, ^8^, ^9^

During a hybrid online and on‐site dermatology conference in Mangalore, India, we conducted a study to assess the impact of an on‐site medical conference on the potential spread of COVID‐19 to the meeting attendants.

## METHODS

The study employed a cohort design and was conducted during the 9th Mid‐annual conference of the Indian Association of Dermatologists, Venereologists, and Leprologists (IADVL), named MIDDERMACON 2021, held in Mangalore on the 28–30 October 2021, after approval from the Institutional Ethics Committee and registration in the Clinical Trials Registry, India.

The conference was held when the Delta variant of SARS‐CoV‐2 was showing a decreasing trend, and the Omicron variant was not yet present. The conference population included delegates, faculty, venue staff, catering staff, and technical and logistic members. The majority of individuals were from India, and four were from the UAE. The majority of attendants were from the states of Karnataka, Kerala, and Maharashtra. The Ministry of Health and Family Welfare (MoHFW) stated that India's COVID‐19 positivity rate on October 28th was 1.42%, 0.42% in Karnataka, and 0.39% in Dakshina Kannada (the district in which the conference took place). However, the neighbouring state of Kerala, the border of which is just 16 km from Mangalore, had a high positivity rate of 10.27%. Maharashtra had a positivity rate of 1.45%. According to Karnataka state guidelines then, attendants from Kerala and Maharashtra were required to provide a negative RT‐PCR COVID‐19 test within 72 h before the meeting. Participants were advised regarding the conference safety protocols, drafted in consideration of the current pandemic (Table 1).

**Table 1:** Advice to all individuals in the conference^a^

• Have received 2 vaccine doses; or 1 vaccine dose if COVID developed later and 2nd could not be completed.
• Do not bring children or other unvaccinated vulnerable population not admitted.
• Do not attend if signs and symptoms of upper respiratory infection are present.
• RT‐PCR test for delegates from Kerala and Maharashtra states.

Abbreviation: RT‐PCR, reverse‐transcription polymerase chain reaction.

^a^ Developed under the supervision of pandemic experts who also provided a meeting protocol. Given the democratic principles, these instructions were issued as ‘Advisory’ instead of ‘Mandatory’.

The scientific sessions in the conference were held in 4 rooms, of which three were of 500 capacity each but were allowed only for 250 individuals. One room was of 1500 capacity, with a maximum of 800 individuals allowed. The delegates were requested not to move around frequently from their seats. All protocols for COVID‐19 were followed, that is, sanitisation at arrival, checking body temperature, wearing N95 masks, access to hand sanitisers, social distancing, keeping doors and windows open for adequate ventilation, and serving food in open spaces. A spacious exhibition centre was provided with space between the stalls, all with a maximum of two staff members allowed. Only 150 delegates were permitted in the exhibition area at any given time.

The study enroled all conference attendants arriving at the front desk willing to participate. The minimum sample size calculated was 493, using a prevalence of 13.3% and a margin error of 3%.^10^ We registered their data, including the mode of travel, vaccination status, and vaccine type, using the web‐based platform Google Forms. All attendants were offered RT‐PCR tests for COVID‐19 infection/carrier status during the 3 days of the conference.

The participants were followed up 15 days after the conference, on the 13 November, 2021, and surveyed regarding COVID‐19 positive status in the previous 15 days via Google Forms. Participants who lived in the area were offered RT‐PCR testing for SARS‐CoV‐2 15 days after the conference.

## RESULTS

The number of delegates and faculty at the conference was 1276. There were 328 co‐delegates, guests, and pharmaceutical company representatives. The number of people in the catering, technical, logistics, and venue staff was 140. Hence, a total of 1744 were physically present at the venue. One hundred ninety‐three individuals participated over a virtual platform.

A total of 576 people responded to our Google Forms survey (response rate of 33.03%). 88.88% (512) of respondents were fully vaccinated, 9.37% (54) were partially vaccinated, and only 1.73% (10) were not vaccinated. Among the vaccinated, 85.06% (490) had taken a viral vector vaccine (Covishield), and 12% (69) had taken an inactivated viral vaccine (Covaxin). An mRNA vaccine (Pfizer) and a viral vector vaccine (Sputnik V) were taken by 0.5% (3) each, while 0.2% (1) took an inactivated viral vaccine (Sinopharm). The majority of the participants took their first vaccine dose between January and June 2021, and the majority of the second doses were between February and October 2021. Eight (1.38%) respondents received booster shots, of which three took Covaxin, three took Covishield, and two took the Pfizer vaccine.

Sixty‐six participants (11.45%) reported having concomitant conditions, of which 31.81% (21) had hypertension, 28.78% (19) diabetes mellitus, 18.18% (12) bronchial asthma, and 1.51% (1) cardiac disease. Most of the 576 participants (61.1%) used collective modes of transportation to reach the conference venue: 36.1% (208) by aeroplane, 14.9% (86) by bus and 10.1% (58) by train. One hundred thirty‐six participants (23.6%) travelled by car, and the rest arrived riding a motorbike or on foot.

Seventy participants reported undergoing a COVID‐19 test 48 h before the conference, and all were negative. During the 3 days of the conference, 120 participants in the group of the 576 respondents accepted to undergo nasopharyngeal and oral swabs for COVID‐19 testing, and none was positive.

Following the conference, 120 local participants accepted to undergo COVID‐19 RT‐PCR sampling, all of which were negative. All 576 conference attendees who had responded earlier filled out new questionnaires 2 weeks after the conference; of these, 5 respondents developed upper respiratory tract infections, of which 1 (0.173%) tested positive for COVID‐19. This person had travelled to the conference by aeroplane and reported only mild symptoms. Hence, the percentage of breakthrough infection after the conference was 0.173% (1/576).

## DISCUSSION

COVID‐19 has forced a break to mass gatherings worldwide, including medical conferences.^11^, ^12^ Travelling and gathering restrictions forced an extended use of web‐based platforms over the past two years. However, online meetings pose difficulties in budget estimations, time management, time‐zone scheduling, and networking.

The MIDDERMACON 2021 meeting, with 1744 individuals meeting on‐site, is the largest on‐site conference held in India since the outbreak of COVID‐19.^13^ The attendants had a vaccination rate similar to other populations of Indian caregivers.^10^ Most of them (85.06%) had received the AstraZeneca vaccine (Covishield) due to its easier availability for the Indian population.

36.1% of attendants travelled by aeroplane to the conference, which has a negligible risk of transmission of infection due to proper screening and mitigation measures.^14^ Breakthrough infection of COVID after the conference was 0.173% (1/576), lower than the 2.605% rate (39/1497) in a study by Bergwerk et al. (which was a hospital‐based study), indicating that the risk of transmission is low when the majority of attendants are vaccinated, and safe preventive measures are followed.^15^ Other reasons for the low rate of infection during the conference may have been the absence of COVID‐19 positive tests in the attendants and the decreasing trend of Delta variant by the time the conference was held. Table 2 summarises the main conclusions of the study (Table 2).

**Table 2:** Main conclusions of the study

• Study conducted at a Dermatology conference during the downward trend of the Delta variant of SARS‐CoV‐2 in coastal South India.
• More than 98% of delegates from India were fully or partly vaccinated; 4 delegates were from the UAE.
• Protocols for COVID–19 prevention were followed, including hand hygiene, social distancing, N95 masks and adequate ventilation.
• Questionnaires were filled by 576 participants, and of these, 120 agreed to undergo a swab test for COVID‐19; none tested positive.
• Participants were followed up after 15 days regarding the occurrence of COVID‐19 infections in the post‐conference period; only 1 participant reported COVID‐19 positivity after the conference out of the 576 included in the study.
• No positive RT‐PCR COVID‐19 tests in 120 local participants tested 2 weeks after the meeting.

Abbreviation: RT‐PCR, reverse‐transcription polymerase chain reaction.

We acknowledge that the reluctance to undergo COVID‐19 swab testing, possibly for fear of testing positive and subsequent quarantine, introduced an unavoidable selection bias, which is the major drawback of our study. Although the results of the questionnaires were kept confidential, it is possible that the reliance on self‐reporting in the questionnaires created a reporting bias, as participants may have been afraid to report symptoms or COVID‐19 positive status.

Our study indicates that on‐site meetings can still be carried out during the COVID‐19 pandemic if vaccination policies are widespread and robust measures for protection and prevention are provided.

## CONFLICT OF INTEREST

The authors declare no conflict of interest.

### ETHICS STATEMENT

All the procedures followed were in accordance with the ethical standards of the Institutional Ethics Committee and with the Helsinki Declaration of 1975, as revised in 1983. CTRI (Clinical Trials Registry) Registration Reference No.: REF/2021/10/048200 and Institutional Ethics Committee Protocol No.: 928/2021.

## Data Availability

The data is collected from the registration desk of IADVL MIDDERMACON 2021. Data used in the study are available upon request from the authors.
